# Cholangioscopy-guided ERCP: expanding diagnostic and therapeutic applications

**DOI:** 10.3389/fmed.2026.1848632

**Published:** 2026-08-03

**Authors:** Shengjie Yang, Hongyu Li

**Affiliations:** Department of Gastroenterology, Changxing County People’s Hospital, Huzhou, Zhejiang, China

**Keywords:** cholangioscopy, difficult bile duct stones, digital single-operator cholangioscopy, direct peroral cholangioscopy, endoscopic retrograde cholangiopancreatography, EyeMax, indeterminate biliary strictures

## Abstract

Endoscopic retrograde cholangiopancreatography (ERCP) remains a cornerstone technique for the management of pancreaticobiliary diseases, but conventional ERCP relies largely on fluoroscopic images and cannot directly evaluate intraductal mucosal abnormalities. Cholangioscopy has expanded the diagnostic and therapeutic scope of ERCP by enabling direct visualization of the biliary tree, targeted tissue acquisition, and visual control of intraductal interventions. The technology has evolved from early dual-operator mother-baby systems to single-operator cholangioscopy, high-resolution digital disposable platforms, direct peroral cholangioscopy using ultraslim endoscopes, and emerging systems such as EyeMax/EYEMAX with larger working channels and improved intraductal access. Clinically, cholangioscopy-guided ERCP is most established for indeterminate biliary strictures and difficult bile duct stones. In indeterminate strictures, cholangioscopy should be interpreted within a multimodality diagnostic framework that includes cross-sectional imaging, conventional ERCP-based sampling, endoscopic ultrasound-guided tissue acquisition, and cholangioscopy-guided visual assessment and biopsy. In difficult stones, cholangioscopy-guided electrohydraulic or laser lithotripsy improves targeted fragmentation and duct clearance when conventional ERCP techniques fail. Emerging applications include assessment of intraductal papillary neoplasms of the bile duct, screening or surveillance of risk-enriched intraductal neoplasms, primary sclerosing cholangitis-associated strictures, post-transplant biliary complications, complex stent guidance, and selected gallbladder interventions. This review summarizes the evolution of cholangioscopy technology, procedural considerations, current evidence, clinical outcomes, safety issues, and future directions including image-enhanced endoscopy and artificial intelligence-assisted image interpretation.

## 1 Introduction

Endoscopic retrograde cholangiopancreatography (ERCP) has evolved from a diagnostic technique into a predominantly therapeutic procedure for biliary and pancreatic ductal diseases. Current ERCP practice includes biliary drainage, stone extraction, stricture management, stent placement, and selected pancreaticobiliary interventions. However, conventional ERCP is limited by its reliance on indirect fluoroscopic imaging. Fluoroscopy depicts ductal anatomy, filling defects, and obstruction, but it does not allow direct inspection of the biliary mucosa (1).

These limitations are clinically important in two common scenarios. First, indeterminate biliary strictures remain difficult to classify as benign or malignant. Conventional ERCP-based tissue acquisition, including brush cytology and fluoroscopy-guided forceps biopsy, has high specificity but limited sensitivity, leading to repeated procedures or delayed diagnosis in some patients (2, 3). Second, difficult bile duct stones, such as large, impacted, intrahepatic, cystic duct confluence, or above-stricture stones, may not be removed using standard sphincterotomy, balloon/basket extraction, large-balloon dilation, or mechanical lithotripsy.

Cholangioscopy addresses these gaps by allowing direct visualization of the bile duct lumen during ERCP. Direct visualization provides assessment of mucosal surface patterns, vascular morphology, papillary or nodular lesions, stone characteristics, ductal anatomy, and the extent of intraductal disease. Cholangioscopy also enables targeted biopsy and visually guided intraductal lithotripsy. As a result, cholangioscopy-guided ERCP has become an important adjunct in modern pancreaticobiliary endoscopy (4–11).

**FIGURE 1:**
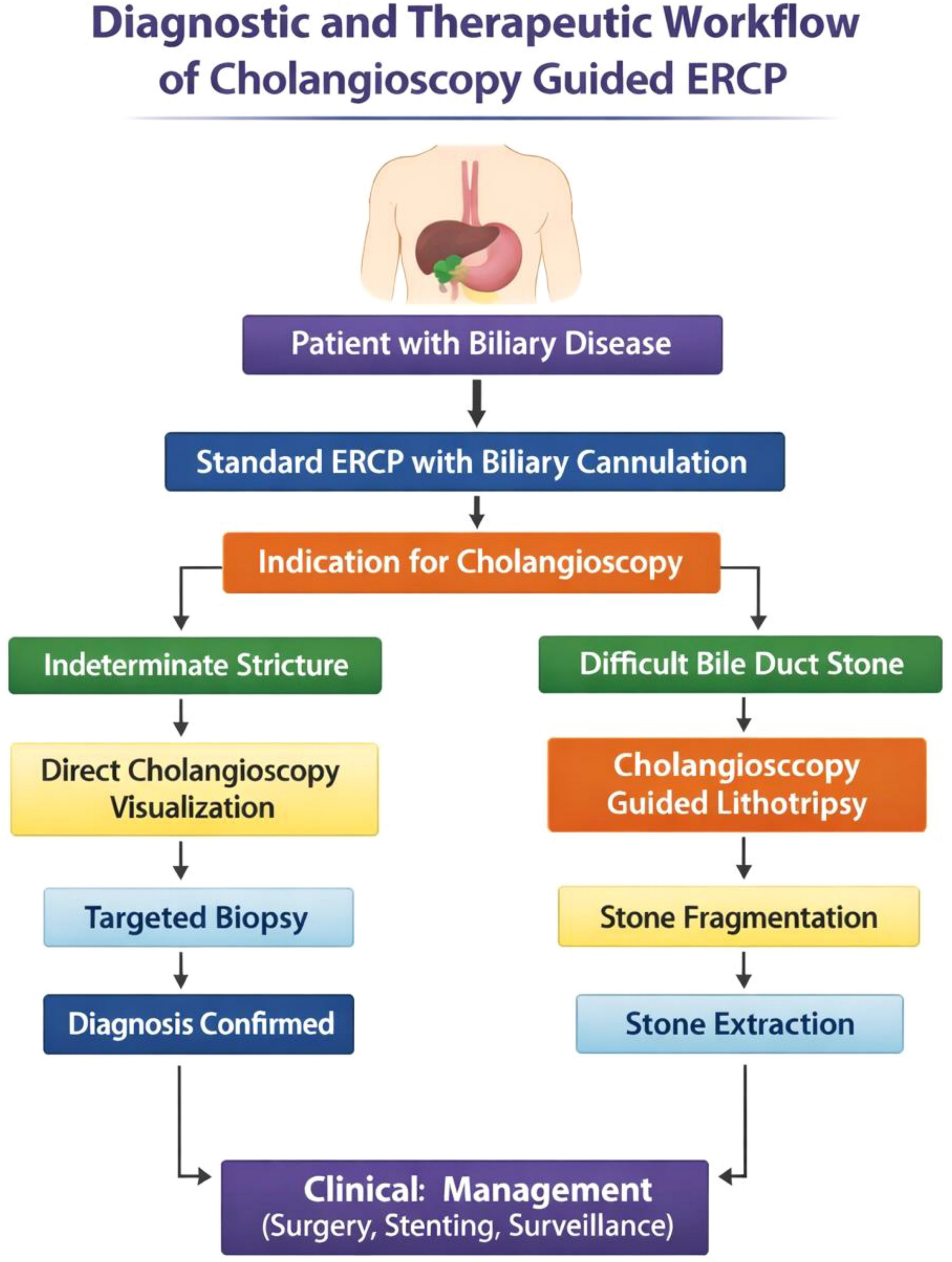
Diagnostic and therapeutic workflow of cholangioscopy-guided ERCP in clinical practice.

In recent years, the field has expanded beyond digital single-operator cholangioscopy. Direct peroral cholangioscopy using ultraslim endoscopes, image-enhanced peroral cholangioscopy with narrow-band imaging, and emerging platforms such as EyeMax/EYEMAX have broadened the technical options and potential indications (7, 8, 12–19). This review summarizes the evolution of cholangioscopy-guided ERCP, compares available platforms, discusses procedural techniques, and reviews current and emerging diagnostic and therapeutic applications. Figure 1 summarizes the diagnostic and therapeutic workflow of cholangioscopy-guided ERCP.

## 2 Evolution of cholangioscopy technology

**FIGURE 2:**
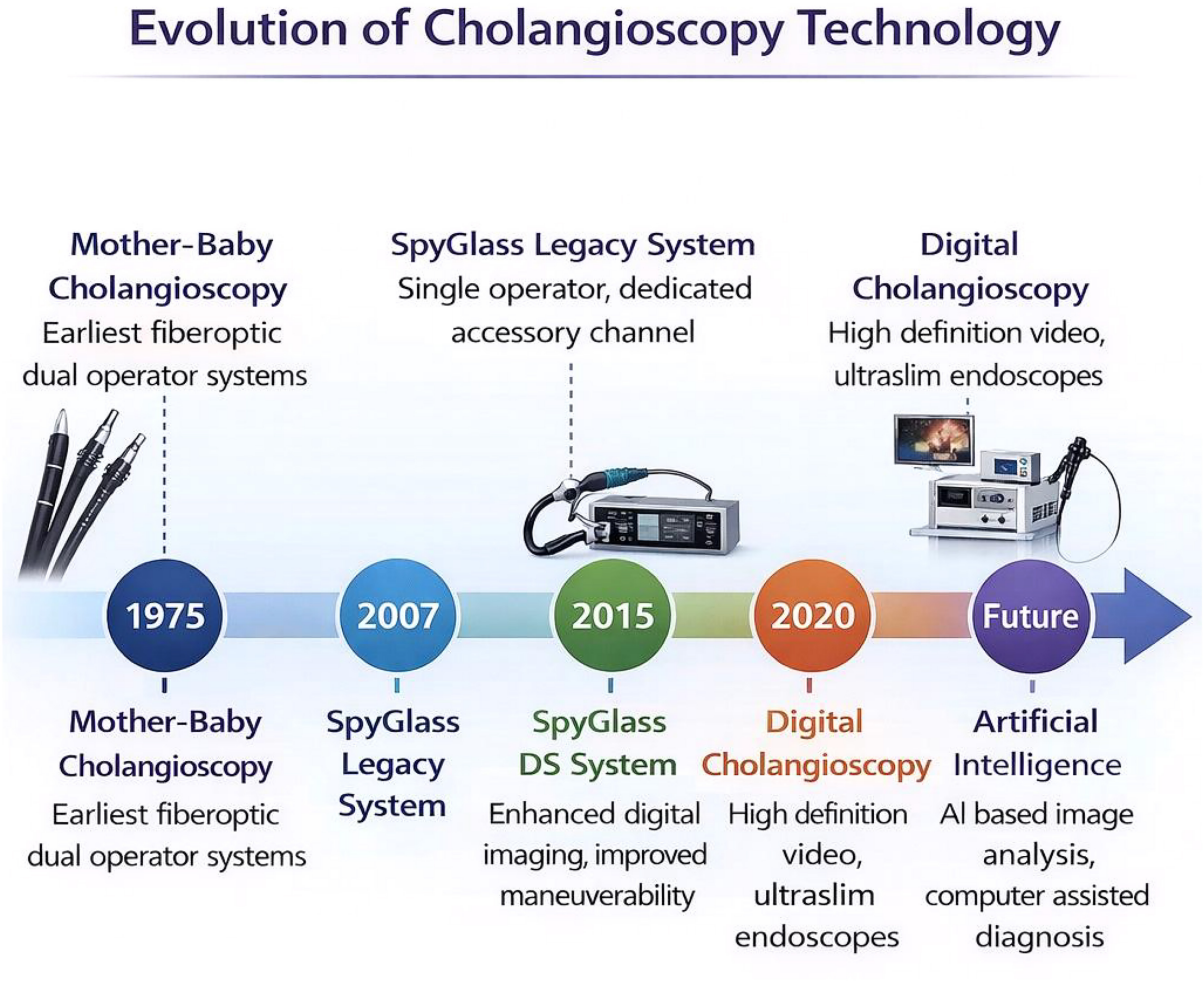
Major milestones in the development of cholangioscopy technology.

Direct visualization of the biliary tract has been a long-standing goal of pancreaticobiliary endoscopy. Early cholangioscopy systems provided proof of concept, but modern clinical adoption became possible only after improvements in scope durability, irrigation, accessory channels, imaging quality, and single-operator control (4–10). Figure 2 summarizes the major milestones, and Table 1 compares current and emerging cholangioscopy platforms.

**TABLE 1:** Major cholangioscopy systems and technical features.

System	Manufacturer	Imaging/approach	Key technical features	Clinical applications	Practical considerations
Mother-baby cholangioscopy	Various early platforms	Fiberoptic	Miniature cholangioscope passed through a therapeutic duodenoscope; required two operators; fragile scope; limited image quality and maneuverability	Historical diagnostic cholangioscopy; selected intraductal biopsy or lithotripsy in expert centers	Two-operator control and fragility limited routine use
SpyGlass Legacy	Boston Scientific	Fiberoptic SOC	Single-operator control, four-way tip deflection, irrigation and accessory channels	Indeterminate strictures, targeted biopsy, difficult stones, intraductal lithotripsy	Improved feasibility but lower image quality than digital systems
SpyGlass DS/DS II	Boston Scientific	Digital disposable SOC	High-resolution digital imaging, disposable catheter, dedicated irrigation, working channel for SpyBite forceps, EHL and laser lithotripsy probes	Indeterminate strictures, difficult stones, PSC strictures, transplant strictures, guidewire/stent assistance	Widely used platform; biopsy samples may remain small
Direct peroral cholangioscopy using ultraslim endoscope	Ultraslim endoscopes from multiple manufacturers	High-definition white-light imaging with possible image enhancement such as NBI	Ultraslim endoscope advanced directly into bile duct after sphincterotomy and/or balloon dilation; larger accessory channel than many disposable SOC systems	Intraductal superficial lesions, IPNB, tumor mapping, targeted biopsy, selected stones and intrahepatic lesions	Technically demanding; limited by duct diameter, scope stability, papillary access, and anatomy
EyeMax/EYEMAX	Micro-Tech/Nanwei Medical Technology	Single-operator direct visualization system	Small outer-diameter models with working channels reported up to approximately 1.8 to 2.0 mm depending on model; designed for biliary visualization, accessories, and stone therapy	Biliary strictures, bile duct stones, cystic duct/gallbladder access in selected reports, emerging biopsy applications	Evidence is emerging; comparative diagnostic studies are ongoing

### 2.1 Early mother-baby cholangioscopy

The earliest cholangioscopy platforms were based on the mother-baby design, in which a small-caliber cholangioscope was advanced through the working channel of a duodenoscope. This configuration enabled intraductal visualization and targeted interventions, but it required two endoscopists, used fragile fiberoptic instruments, and offered limited image quality and maneuverability (4, 6). These limitations restricted widespread adoption despite the conceptual advantage of direct biliary visualization.

### 2.2 Single-operator cholangioscopy and digital platforms

The development of single-operator cholangioscopy (SOC) represented a major practical advance. The SpyGlass Direct Visualization System allowed one endoscopist to control both the duodenoscope and cholangioscope while using dedicated irrigation, accessory, and biopsy channels. The first-generation platform was fiberoptic, whereas the later SpyGlass DS and DS II platforms introduced digital imaging, improved image quality, four-way tip deflection, and disposable catheters compatible with biopsy forceps and lithotripsy probes (5, 9, 10). Digital SOC has therefore become the most widely used cholangioscopy platform in many tertiary centers.

### 2.3 Direct peroral cholangioscopy using ultraslim endoscopes

Direct peroral cholangioscopy (D-POC) using an ultraslim upper endoscope is an alternative approach in which the endoscope itself is advanced directly through the papilla into the bile duct, usually after sphincterotomy and/or papillary balloon dilation. This technique can provide high-definition white-light imaging, narrow-band imaging, a larger working channel than many disposable cholangioscopes, and the possibility of using standard biopsy devices in selected cases (7, 8, 12–16). These advantages make D-POC attractive for assessing intraductal superficial lesions, intraductal papillary neoplasms of the bile duct (IPNB), longitudinal tumor spread, and selected stone disease. However, D-POC is technically demanding because scope stability, looping in the stomach or duodenum, papillary access, bile duct diameter, and patient anatomy can limit successful intraductal advancement. Therefore, D-POC is generally reserved for centers with appropriate expertise and selected indications.

### 2.4 EyeMax/EYEMAX and next-generation cholangioscopy systems

EyeMax/EYEMAX has been introduced as an emerging single-operator cholangioscopy system designed for direct intraductal visualization, bile duct stone management, and evaluation of biliary strictures. Published descriptions report EyeMax models with small outer diameters and working channels that may be larger than those of some earlier disposable cholangioscopy systems, potentially allowing improved irrigation and accessory use (18). Early clinical experience has described ERCP plus EyeMax for choledocholithiasis-associated acute cholecystitis, with reported technical and clinical success rates of 92% and 96%, respectively, in a 25-patient series (18). A multicenter randomized study comparing EYEMAX with SpyGlass DS for biliary stricture biopsy quality and diagnostic yield has also been registered, reflecting increasing interest in this platform (19).

### 2.5 Expansion of indications

The initial indications for cholangioscopy were diagnosis of indeterminate biliary strictures and management of difficult stones. Recent literature supports a broader role, including assessment of intraductal superficial lesions, IPNB and other intraductal neoplasms of the bile duct, mapping of tumor extension, primary sclerosing cholangitis-related strictures, post-transplant biliary complications, cystic duct confluence stones, gallbladder access in selected cases, complex guidewire placement, and stent optimization (8, 12–18, 20–25).

## 3 Procedural techniques of cholangioscopy-guided ERCP

Cholangioscopy-guided ERCP begins with standard ERCP techniques. The papilla is reached with a therapeutic duodenoscope, selective biliary cannulation is obtained, and cholangiography is performed to define ductal anatomy, strictures, filling defects, stones, leaks, or stent-related findings. Guidewire-assisted cannulation, careful contrast injection, and risk-adapted prophylaxis for post-ERCP pancreatitis should be used according to guideline-based ERCP practice (1, 26, 27).

### 3.1 Biliary access and preparation for cholangioscopy

After biliary cannulation, sphincterotomy and/or papillary dilation may be performed when needed to facilitate cholangioscope insertion, improve drainage, or permit stone therapy. The decision should consider indication, coagulation status, papillary anatomy, duct diameter, stone size, and the need for repeat access. For disposable SOC systems, the cholangioscope is advanced through the duodenoscope over or alongside a guidewire. For D-POC using an ultraslim endoscope, papillary dilation and overtube or anchoring methods may be required to maintain scope stability (7, 8).

### 3.2 Intraductal visualization and irrigation

Continuous or intermittent saline irrigation is used to clear bile, blood, sludge, or debris. Visualization should be balanced against the risk of increasing intraductal pressure, particularly in obstructed systems. Adequate biliary drainage and selective antibiotic prophylaxis are important when drainage is incomplete or prolonged cholangioscopy with irrigation is expected (19).

### 3.3 Targeted biopsy and tissue acquisition

Targeted biopsy is performed by advancing miniature forceps through the cholangioscope working channel to suspicious mucosa, nodular lesions, papillary projections, or areas with abnormal vascularity. Because cholangioscopy-guided biopsy specimens are small, multiple biopsies should be obtained when feasible. Tissue adequacy may be improved by careful targeting, repeated sampling, and, where available, larger-channel platforms or direct peroral cholangioscopy that permits larger biopsy forceps (8, 20, 21, 23, 24).

### 3.4 Cholangioscopy-guided therapy

Therapeutic cholangioscopy is most commonly used for difficult bile duct stones. Electrohydraulic lithotripsy and laser lithotripsy can be applied under direct visualization to fragment stones while minimizing injury to the duct wall. Cholangioscopy may also assist guidewire placement across complex strictures, selective ductal access, stent evaluation, and intraductal treatment planning (25, 28–32).

## 4 Clinical applications of cholangioscopy-guided ERCP

]Tables 2, 3 summarize the diagnostic approaches for indeterminate biliary strictures and the clinical outcomes of cholangioscopy-guided lithotripsy, respectively.

**TABLE 2:** Diagnostic approaches for indeterminate biliary strictures.

Approach	Strengths	Limitations	Representative performance/evidence	Best clinical role
Brush cytology during ERCP	Simple, low-cost, widely available; can be performed during index biliary drainage	Limited sensitivity; false negatives common	Pooled sensitivity approximately 45% for malignant biliary strictures; specificity high	Initial ERCP sampling, especially when drainage is needed
Fluoroscopy-guided forceps biopsy during ERCP	Provides tissue architecture; improves yield when combined with brushing	Sampling remains indirect; may miss eccentric or intraductal mucosal lesions	Combination of brushing and biopsy improves sensitivity compared with either alone	Index ERCP sampling; recommended where technically feasible, especially perihilar strictures
EUS-guided tissue acquisition	High yield for distal strictures, pancreatic masses, lymph nodes, and extraluminal disease; provides staging information	May be less useful for purely intraductal mucosal lesions; caution in potentially resectable perihilar disease	Guidelines support EUS-TA plus ERCP-TA for jaundiced distal extrahepatic strictures without pancreatic mass	Distal strictures, mass-forming disease, lymphadenopathy, staging, nondiagnostic ERCP sampling
POCS visual impression	Direct evaluation of mucosal and vascular patterns; immediate intraprocedural assessment	Operator dependent; visual-pathology discrepancy can occur	Pooled sensitivity/specificity of visual impression reported around 84.5%/82.6% in earlier meta-analysis	Intraductal mucosal lesions, suspected malignancy, mapping of tumor extent
POCS-guided targeted biopsy	Targets suspicious areas directly; higher yield after nondiagnostic conventional sampling	Small tissue samples; false negatives possible; cost and expertise required	Pooled sensitivity/specificity reported around 60.1%/98.0% overall; 74.7%/93.3% after prior negative brushing/biopsy in selected studies; DSOC meta-analysis reported sensitivity 0.74 and specificity 0.98	Persistent indeterminate strictures, perihilar strictures, intraductal lesions, nondiagnostic ERCP/EUS results
NBI-enhanced direct POC	Improves visualization of surface and microvascular patterns	Requires direct POC expertise and adequate ductal access	Recent studies support NBI for predicting malignancy and classifying intraductal superficial lesions	Selected centers; suspected intraductal superficial lesions or IPNB
Multimodality strategy	Combines complementary strengths of imaging, ERCP sampling, EUS-TA, and POCS-guided biopsy	Requires coordination, expertise, and individualized sequencing	ESGE guideline supports standard ERCP modalities at index ERCP and cholangioscopy-guided biopsy for indeterminate strictures; EUS-TA emphasized for distal/mass-forming disease	Most clinically relevant approach when diagnosis remains uncertain or management depends on tissue confirmation

**TABLE 3:** Clinical outcomes of cholangioscopy-guided lithotripsy for difficult bile duct stones.

Study	Year	Design	Technique	Stone clearance or success	Key finding
Maydeo et al. (28)	2011	Prospective single-center study	SOC-guided laser lithotripsy	Approximately 90%	Established feasibility and high success of SOC-guided laser lithotripsy for difficult biliary and pancreatic ductal stones
Bhandari et al. (29)	2016	Systematic review/meta-analysis	Cholangioscopy-guided lithotripsy using EHL or laser	Approximately 88% to 95% across included studies	Supported high clearance rates in complex stones but noted heterogeneity among studies
Buxbaum et al. (30)	2018	Randomized trial	Cholangioscopy-guided laser lithotripsy vs. conventional therapy	93% vs. 67%	Laser lithotripsy improved duct clearance but increased procedure time
Galetti et al. (31)	2020	Systematic review/meta-analysis	Cholangioscopy-guided lithotripsy vs. conventional therapy	88.29% pooled clearance; 72.7% first-session clearance	Adverse event rate about 8.7%; conventional therapy had shorter procedure time
Miyamoto et al. (25)	2026	Retrospective cohort	POCS-guided lithotripsy for cystic duct confluence stones when needed	100% complete stone removal; POCS-guided lithotripsy needed in 50%	Stone minor axis/distal duct diameter > 1.2 predicted need for POCS-guided lithotripsy
Tao et al. (18)	2023	Early clinical series	ERCP plus EyeMax SOC for acute cholecystitis secondary to choledocholithiasis	Technical success 92%; clinical success 96%	Suggested feasibility of EyeMax-based SOC for selected gallbladder/biliary stone applications

### 4.1 Indeterminate biliary strictures: comparison with EUS-guided tissue acquisition, ERCP sampling, and multimodality diagnosis

Indeterminate biliary strictures are among the most important indications for cholangioscopy. A stricture is generally considered indeterminate when cross-sectional imaging, laboratory evaluation, and standard ERCP sampling do not establish a definitive benign or malignant diagnosis. Malignancy may be suggested by irregular mucosa, nodularity, papillary projections, ulceration, neovascularization, dilated tortuous vessels, and friability, whereas benign inflammatory strictures more often show smooth mucosa and regular vascular patterns. However, visual impression alone is operator dependent and should usually be complemented by tissue acquisition (11, 23, 24).

Conventional ERCP-based sampling remains important because it can be performed during the index drainage procedure. Brush cytology is simple and highly specific but has limited sensitivity. Fluoroscopy-guided forceps biopsy can increase yield, and the combination of brushing plus biopsy is preferred over brushing alone when technically feasible, particularly for perihilar strictures (2, 3, 20, 22). Molecular adjuncts such as fluorescence in situ hybridization or next-generation sequencing may improve diagnostic confidence where available, but they are not universally accessible.

EUS-guided tissue acquisition (EUS-TA) is complementary rather than competitive. EUS-TA is particularly useful for distal biliary strictures, pancreatic masses, enlarged lymph nodes, or extraluminal lesions. It can also provide staging information. In perihilar strictures, EUS-TA requires caution when curative surgery or transplantation is being considered because of potential concerns about tumor seeding and because intraductal mapping may be more clinically relevant. Recent guidance recommends combining EUS-TA and ERCP-based tissue acquisition for jaundiced distal extrahepatic strictures without a pancreatic mass and using cholangioscopy-guided biopsy after standard ERCP modalities when the diagnosis remains indeterminate (20, 21).

Cholangioscopy contributes in two ways: it enables direct visual characterization and allows targeted biopsy from abnormal mucosa. Its value is greatest when disease is primarily intraductal, when strictures are proximal/perihilar, when previous ERCP sampling is nondiagnostic, or when assessment of tumor extent is required. Nevertheless, cholangioscopy-guided biopsy can still have false-negative results because tissue samples are small and some strictures are submucosal or extrinsic. Therefore, a multimodality strategy is often necessary. In practical terms, cross-sectional imaging and clinical assessment should be followed by ERCP drainage with brushing and biopsy when indicated; EUS-TA should be added for distal or mass-forming disease; and cholangioscopy should be used for persistent indeterminate strictures, perihilar disease, intraductal lesions, or when targeted visual biopsy is expected to change management (20, 21).

### 4.2 Difficult bile duct stones and intraductal lithotripsy

Most common bile duct stones can be removed with conventional ERCP techniques. Cholangioscopy becomes valuable when stones are large, impacted, multiple, located above strictures, located in intrahepatic ducts, associated with altered anatomy, or have failed previous extraction attempts. Under direct visualization, electrohydraulic lithotripsy (EHL) and laser lithotripsy can be targeted precisely to the stone surface, avoiding blind energy delivery to the duct wall (25, 28–32).

Evidence supports high rates of stone clearance. A randomized trial showed higher clearance with cholangioscopy-guided laser lithotripsy than conventional therapy for large bile duct stones, although procedure time was longer (30). Meta-analyses report overall duct clearance rates around 88% to 91% and first-session clearance rates around 73% to 77%, depending on included populations and techniques (29, 31). Recent data also support a role in cystic duct confluence stones. In a Japanese retrospective study, complete stone removal was achieved in all patients, but POCS-guided lithotripsy was required in half of cases, and a stone-to-distal bile duct diameter ratio greater than 1.2 predicted the need for POCS-guided lithotripsy (25).

### 4.3 Intraductal tumors and intraductal papillary neoplasms of the bile duct

Cholangioscopy is increasingly used for intraductal tumors, including IPNB, superficial cholangiocarcinoma, and other intraductal neoplasms of the bile duct. These lesions may be missed or underestimated by cholangiography because they can appear as subtle mucosal abnormalities, papillary projections, or superficial spreading lesions. Direct visualization helps detect lesions, define longitudinal extent, guide biopsy, and plan surgical margins (13–17).

Direct POC with narrow-band imaging has been studied for detection and classification of intraductal neoplasms. A large study of direct POC with NBI proposed an endoscopic classification based on surface structure and microvascular patterns (14). A more recent comparison of disposable digital SOC versus direct POC found both approaches clinically relevant for intraductal superficial lesions, while highlighting that platform selection may depend on duct access, image-enhancement capability, working channel, and procedural expertise (15). Recent studies also support the use of POCS for IPNB diagnosis and management and for risk-enriched detection and surveillance of intraductal neoplasms after bile duct stone removal (16, 17).

### 4.4 Other established and emerging applications

Other applications include evaluation of dominant strictures in primary sclerosing cholangitis, assessment of post-liver transplant biliary strictures, selective guidewire placement across complex strictures, evaluation of stent occlusion or tumor ingrowth, and confirmation of complete stone clearance. In selected centers, cholangioscopy may also facilitate gallbladder access through the cystic duct, although this remains technically demanding and should be considered investigational or highly selective (8, 11, 18).

## 5 Diagnostic accuracy and clinical outcomes

The diagnostic performance of cholangioscopy depends on the indication, platform, endoscopist experience, sampling technique, lesion location, and reference standard. For indeterminate biliary strictures, cholangioscopy visual impression tends to be sensitive but less specific than histologic confirmation, whereas targeted biopsy provides high specificity but only moderate sensitivity because of small samples and submucosal or extrinsic disease (23, 24). Digital SOC meta-analysis has reported pooled sensitivity of 0.74 and specificity of 0.98 for targeted biopsy in indeterminate biliary strictures (24).

A clinically useful interpretation is that cholangioscopy is not a replacement for conventional sampling or EUS-TA, but rather a targeted adjunct when standard approaches are inadequate or when intraductal disease is suspected. For distal strictures with a pancreatic mass or extraluminal lesion, EUS-TA often provides a more efficient diagnosis and staging information. For perihilar strictures, intraductal superficial lesions, IPNB, or persistent negative sampling despite high suspicion, cholangioscopy-guided visual assessment and biopsy are particularly valuable (20, 21).

For difficult bile duct stones, clinical outcomes are more consistently favorable. Cholangioscopy-guided lithotripsy provides direct visualization of stone fragmentation and helps avoid surgery in many patients after failed conventional ERCP. However, the approach may require longer procedure time, specialized equipment, and expertise (30, 31).

## 6 Safety, adverse events, and limitations

The adverse event profile of cholangioscopy-guided ERCP overlaps with standard ERCP and includes post-ERCP pancreatitis, cholangitis, bleeding, perforation, and cardiopulmonary events related to sedation. Additional considerations include prolonged procedure time, intraductal irrigation, increased intraductal pressure, and complex therapeutic maneuvers. Risk mitigation includes careful patient selection, prophylaxis for post-ERCP pancreatitis when indicated, antibiotic prophylaxis when biliary drainage may be incomplete, limiting irrigation pressure, and ensuring adequate drainage at the end of the procedure (1, 27).

Cost and availability are important limitations. Disposable digital cholangioscopes and dedicated accessories can be expensive, and the procedure requires training and institutional support. Diagnostic interpretation can be operator dependent, and biopsy specimens may be small. Direct POC using ultraslim endoscopes can offer better image enhancement and larger channels, but it has a steeper technical learning curve and may not be feasible in nondilated ducts or unstable access (8). EyeMax/EYEMAX and other newer platforms may address some limitations, but comparative data remain limited and further prospective studies are needed (18, 19).

## 7 Future perspectives and emerging technologies

### 7.1 Image-enhanced cholangioscopy

Image-enhanced endoscopy, especially narrow-band imaging during direct POC, may improve detection of mucosal and vascular patterns associated with dysplasia or malignancy. Recent studies evaluating NBI during POC for indeterminate strictures and intraductal neoplasms suggest that image-enhancement may improve lesion characterization, but broad adoption will require validation, standardized classification systems, and training (12, 14).

### 7.2 Artificial intelligence-assisted image interpretation

Artificial intelligence may reduce interobserver variability and support real-time classification of benign and malignant biliary findings. Pilot work using cholangioscopy images has shown promising diagnostic performance, but larger multicenter datasets, external validation, and integration with clinical variables are necessary before routine use (33, 34).

### 7.3 Platform innovation and precision biliary endoscopy

Next-generation cholangioscopy platforms are expected to improve image resolution, irrigation, deflection, working-channel diameter, compatibility with accessories, and cost-effectiveness. Larger working channels may improve biopsy adequacy and therapeutic capability. Platform choice may increasingly be tailored to the clinical problem: disposable SOC for efficient standard intraductal evaluation and lithotripsy; direct POC for high-definition/NBI assessment of superficial lesions; and emerging systems such as EyeMax/EYEMAX for selected diagnostic and therapeutic applications (16, 18, 19).

## 8 Conclusion

Cholangioscopy-guided ERCP has evolved from fragile dual-operator fiberoptic systems into a versatile set of technologies that includes digital single-operator platforms, direct peroral cholangioscopy with ultraslim endoscopes, and emerging systems such as EyeMax/EYEMAX. These advances have expanded ERCP from fluoroscopy-guided intervention to direct intraductal diagnosis and therapy. The strongest clinical roles are the evaluation of indeterminate biliary strictures and management of difficult bile duct stones. In biliary strictures, cholangioscopy is most useful as part of a multimodality diagnostic pathway that integrates conventional ERCP sampling, EUS-guided tissue acquisition, imaging, and clinical context. In difficult stones, cholangioscopy-guided lithotripsy improves targeted fragmentation and duct clearance after failed conventional ERCP. New indications, improved platforms, image-enhanced endoscopy, and artificial intelligence may further expand the role of cholangioscopy in precision pancreaticobiliary endoscopy.
